# A Brain-Controlled and User-Centered Intelligent Wheelchair: A Feasibility Study

**DOI:** 10.3390/s24103000

**Published:** 2024-05-09

**Authors:** Xun Zhang, Jiaxing Li, Ruijie Zhang, Tao Liu

**Affiliations:** 1 School of Mechanical Science and Engineering, Northeast Petroleum University, No. 99 Xuefu Street, Longfeng District, Daqing 163319, China; 218004041413@stu.nepu.edu.cn (X.Z.); 261974020133@nepu.edu.cn (R.Z.); 2 The State Key Laboratory of Fluid Power and Mechatronic Systems, School of Mechanical Engineering, Zhejiang University, Hangzhou 310058, China; 12225069@zju.edu.cn

**Keywords:** intelligent wheelchair, UCD, ergonomics, multi-posture, design, brain–computer interfaces

## Abstract

Recently, due to physical aging, diseases, accidents, and other factors, the population with lower limb disabilities has been increasing, and there is consequently a growing demand for wheelchair products. Modern product design tends to be more intelligent and multi-functional than in the past, with the popularization of intelligent concepts. This supports the design of a new, fully functional, intelligent wheelchair that can assist people with lower limb disabilities in their day-to-day life. Based on the UCD (user-centered design) concept, this study focused on the needs of people with lower limb disabilities. Accordingly, the demand for different functions of intelligent wheelchair products was studied through a questionnaire survey, interview survey, literature review, expert consultation, etc., and the function and appearance of the intelligent wheelchair were then defined. A brain–machine interface system was developed for controlling the motion of the intelligent wheelchair, catering to the needs of disabled individuals. Furthermore, ergonomics theory was used as a guide to determine the size of the intelligent wheelchair seat, and eventually, a new intelligent wheelchair with the features of climbing stairs, posture adjustment, seat elevation, easy interaction, etc., was developed. This paper provides a reference for the design upgrade of the subsequently developed intelligent wheelchair products.

## 1. Introduction

According to statistics from the National Bureau of Statistics, by the end of 2021, there were 24.72 million people with physical disabilities in China, and the number of elderly people aged 65 and above exceeded 200 million. From here, the demand for intelligent wheelchairs that assist in mobility for people with motor disabilities will only continue to increase. People with lower limb disabilities typically face extraordinary obstacles in independent travel because of their physiological defects, and the lack of accessible facilities in much of the world further exacerbates how they are inconvenienced. In this context, an intelligent wheelchair that allow users to travel up and down stairs and over rough terrain would constitute a practical, intelligent assistive product for people with lower limb disabilities. The development of chip manufacturing, artificial intelligence, and Internet of Things technology provides the technological basis for the intelligence of wheelchair products. Compared with ordinary electric wheelchairs, the intelligent wheelchair combines an environment perception system, embedded control system (PC, DSP controller), drive system, and a variety of human–computer interaction modules [1]. Various sensor modules and tailored structural designs diversify the functions of the intelligent wheelchair and, in doing so, reduce the dependence of people with lower limb disabilities on their caregivers.

Although some advanced wheelchair technologies have already achieved functionalities such as autonomous navigation, obstacle avoidance, and voice control, these capabilities still fall short of meeting the needs of severely disabled individuals who have almost lost their mobility. Despite the loss of physical abilities, their brain functions remain largely intact, similar to those of able-bodied individuals. Therefore, harnessing the brain’s electroencephalographic (EEG) signals to control high-performance intelligent wheelchairs is imperative. Designing a brain-controlled intelligent wheelchair is crucial as it can provide greater mobility autonomy for severely disabled individuals while ensuring their safety.

## 2. Research Background

### 2.1. Paradigm of the Brain–Computer Interface for Intelligent Device Control

In 1924, German psychiatrist Hans Berger first recorded human brain waves, thus initiating the study of brain electrical signals [2]. In 1973, Jacques Vidal and others from the United States invented a system of using an EEG to control a computer cursor for two-dimensional movement. Subsequently, they published the first paper on brain–computer interfaces [3]. The U.S. Department of Health’s Wadsworth Research Center designed and developed a free, universal brain–computer interface software system called BCI2000. Using BCI2000, they designed a character input system for a completely paralyzed patient [4,5]. The BCI research group at Graz University of Technology in Austria proposed BCI technology based on actual and imagined movements, combined with virtual reality technology applied in disability rehabilitation [6,7]. Gao Shangkai at Tsinghua University designed a real-time telephone dialing system based on SSVEP, and used motor imagery BCI to control robotic dogs playing soccer, among other applications [8,9]. The Technical University of Berlin in Germany implemented character input systems called Hex-o-spell and a pinball machine device based on two different types of motor imagery tasks [10,11,12].

The paradigm of the brain–computer interface (BCI) can be categorized into two main types: spontaneous and evoked. Spontaneous BCI refers to rhythmic electrical potential changes in the brain cortex that occur spontaneously without specific external stimuli. A common example is the motor imagery (MI) paradigm, where individuals generate EEG signals associated with imagined movements, such as imagining moving a limb without actually doing so. Leeb et al. conducted experiments where paralyzed participants imagined tasks like clenching their left hand, squeezing their right hand, and walking, to control the directional movements of a wheelchair [13]. While the MI paradigm offers rapid response times and real-time control, it demands high user restrictions, exhibits poor individual adaptability, and requires extensive training, with limited selection of commands for controlling the system.

The evoked potential paradigm refers to changes in brain electrical potentials in certain brain regions when specific external stimuli are received, known as event-related potentials (ERPs). Visual stimulation is the most extensively studied, with commonly used paradigms including P300 and steady-state visual evoked potentials (SSVEPs). The P300 potential typically appears at around 300 ms after relevant stimuli, presenting as a positive peak in EEG recordings, with the signal becoming more prominent when participants receive low-probability stimuli. He et al. employed a visual interface with a target button and three distractors appearing randomly, allowing participants to switch commands by focusing on the target button to control the wheelchair [14]. The P300 paradigm can be induced with short training periods and offers stability but demands high user concentration and may be easily affected by fatigue. Steady-state visual evoked potentials typically focus on the occipital region of the brain and are elicited by flickering visual stimuli at frequencies greater than 6 Hz, inducing brain electrical signals at the same frequency or its harmonics. Mistry et al. used LED to present four stimulation targets and calculated SSVEP peak amplitudes as thresholds for discrimination, used to control the wheelchair’s movement direction [15]. Experimental designs based on SSVEP are straightforward, require minimal user training, exhibit strong individual adaptability, and have become a focal point in BCI research.

### 2.2. Intelligent Wheelchair

The intelligent wheelchair has some unique advantages compared to other intelligent devices for assisting individuals with mobility impairments, such as exoskeletons, robotic arms, and humanoid robots. The intelligent wheelchair is easy to operate, with many featuring automatic cruise control functions, allowing users to control the wheelchair’s movement through simple manipulation. This also makes the intelligent wheelchair more suitable for brain–computer interfaces with fewer commands. The intelligent wheelchair is customizable to meet individual user needs, with options for seat adjustment, personalized control settings, and the addition or removal of extra features, ensuring better adaptation to the specific requirements of different users. Furthermore, the intelligent wheelchair is more suitable for prolonged use compared to other devices, offering high levels of comfort.

Since the world’s first intelligent wheelchair was invented by the British in 1986, researchers have been working on intelligent wheelchair development for nearly 40 years. In 1995, the TINMAN project at the KISS Institute in the United States adopted a hierarchical control architecture in its intelligent wheelchair system. It designed three manual control modes for human–machine interaction and included basic autonomous obstacle avoidance capabilities [16]. In 1998, the WHEELESLEY intelligent wheelchair developed by MIT used a computer as the control core, employed ultrasonic sensors, and utilized a laptop as the human–machine interaction device for wheelchair control. Its main features included semi-autonomous obstacle avoidance and collision prevention [17]. Also in 1998, France developed the VAHM intelligent wheelchair, using a PC486 as the controller and ultrasonic sensors to achieve autonomous navigation and obstacle avoidance. The second-generation VAHM wheelchair improved the human–machine interaction mode, expanding the range of use for wheelchair users [18]. In 1998, a Greek research institution designed the SENARIO intelligent wheelchair, focusing on auxiliary sensor technology to enhance human–machine interaction while achieving high-performance semi-autonomous and autonomous navigation functions [19]. In 1999, the University of Ulm in Germany focused on autonomous navigation and developed the Maid intelligent wheelchair, solving autonomous navigation and obstacle avoidance challenges in complex train station environments [20]. In 2001, a Spanish research institution developed the SIAMO intelligent wheelchair, which featured a distributed system structure tailored to the varying degrees of disability among users. It incorporated multiple interface devices such as visual, infrared, and voice, significantly enriching the human–machine interaction and laying a solid foundation for the commercialization of intelligent wheelchairs [21]. Japanese scholars designed an intelligent wheelchair controlled based on user head posture and eye state. This wheelchair utilized visual sensors to gather information on the user’s head and eye movements, achieving wheelchair control based on user intention through data fusion processing [22]. The Canadian company AAI further integrated GPS (Global Positioning System) and RFID (Radio Frequency Identification) into the TAO intelligent wheelchair system to assist in autonomous navigation, significantly improving control accuracy and safety [23]. Japan shows an advanced design concept in intelligent wheelchair products, which highlights the emotional needs of people with lower limb disabilities and places emotional and humanized design in the early research stage [24]. WHILL Technology partnered with Panasonic Electronics to launch the WHILLNEXT, an intelligent wheelchair robot capable of autonomous driving, which was first tested at Haneda Airport in Japan in 2018. WHILLNEXT is equipped with a map and navigation system, users can enter a location via a smartphone, and the intelligent wheelchair will plan the optimized route and drive to the destination. The TOPCHAIRS intelligent wheelchair released by Top chair Technology in France made a breakthrough in appearance design and function. The appearance design of this wheelchair highlights the functionalist design concept. In addition to detecting and crossing obstacles in the surrounding environment, this wheelchair can also safely go up and down the stairs. After switching between the wheels and tracks through the button, TOPCHAIRS will automatically detect the beginning and end of the stairs. At the same time, the TOPCHAIRS intelligent wheelchair can also transform into a storage form through a change in mechanical structure, which makes it more convenient to carry and saves transportation space.

Among the intelligent wheelchair products that are independently designed in China, the “Jiao long” intelligent wheelchair developed by Shanghai Jiao Tong University is most representative. The innovation point of this wheelchair lies in a variety of interaction forms that mean users can control the wheelchair by voice interaction. The corresponding function can be achieved by giving oral commands to the intelligent wheelchair and this function is still being further optimized. Another representative intelligent wheelchair, the “Robot Chair” intelligent wheelchair, is developed by the Institute of Automation, Chinese Academy of Sciences. The breakthrough point of “Robot Chair” is the new human–machine engineering interface that means users can control the wheelchair through brain waves, electromechanical tools, and other human–machine interfaces. This wheelchair focuses on the human–machine interface and interaction form, giving less priority to the structure and appearance CMF design.

At present, the main research direction of intelligent wheelchairs in China is to achieve new human–computer interaction methods and better wheelchair functions, rather than practical structural design. Very limited consideration is given to the acceptance of consumers with regard to the product appearance and texture, and there are few market-oriented products. In addition, many wheelchairs on the market, which claim to be “intelligent wheelchairs” in their product naming, have not achieved intelligence and humanization in their function and design. These products provide a single function and are very complex in terms of direction and operation, which is difficult for users to accept. At the same time, there are also many shortcomings in emotional, humanized, and safety design. In terms of appearance, the current design of intelligent wheelchairs in China is simple and crude, just a simple stacking of mechanical structures.

With the advancement of technology and the increasing performance requirements of users for products, it seems that the future development trends of intelligent wheelchairs will involve the Internet of Things, an ecological focus, artificial intelligence, high-end CMF (Color–Material–Finishing) design, and high-end customized design.

### 2.3. Brain-Controlled Wheelchair

The current research focus is on the diversity of human–machine interaction methods, particularly the development of multimodal control methods to maximize the adaptability of wheelchairs for different users’ needs. In the UK, Chun S. and colleagues proposed an intelligent wheelchair controlled by electromyography (EMG) [25,26]. This type of intelligent wheelchair utilizes EMG sensors to capture EMG signals from the user’s facial muscles, which are then processed and analyzed to generate wheelchair directional control commands for wheelchair control. In 2007, Japan developed an intelligent wheelchair controlled using speech recognition technology [27]. Indonesian and Japanese scholars used visual sensors to capture dynamic data information from the user’s eyes and eyelids, enabling control of the intelligent wheelchair [28]. Japanese scholars achieved control of an intelligent wheelchair combining electromyography and eye-tracking EEG [29], further expanding the range of human–machine interaction utilizing EMG signals. The RIKEN Brain Science Institute (BSI) and Toyota Collaboration Center in Japan subsequently developed intelligent wheelchairs controlled via BCI [30,31,32]. Users wear sensors to detect brainwave signals and can automatically adjust parameters through self-learning to accommodate different operators. The accuracy of operation exceeds 95%, achieving the goal of controlling the intelligent wheelchair through conscious thought and advancing the wheelchair towards greater intelligence.

Conventional intelligent wheelchairs are relatively easy to operate but still require some level of limb control, posing challenges for individuals with severe motor disabilities. Brain-controlled wheelchairs (BCWs) offer a promising solution by eliminating the need for limb involvement. Utilizing brain–computer interfaces (BCIs), a BCW establishes a direct connection between the brain and the wheelchair, enabling control through brain signals and overcoming the physical limitations of conventional wheelchairs.

Tanaka et al. developed the first EEG wheelchair, utilizing a novel recursive training algorithm to identify EEG signals for motion control [33]. This enabled the control of an electric wheelchair through EEG signals, as demonstrated in test results. Turnip et al. proposed an adaptive neuro-fuzzy interference system classifier for SSVEP recognition to control an electronic wheelchair, achieving over 80% accuracy. Surei Mouli et al. pioneered an in-ear electrode for SSVEP data collection, expanding the wearable capabilities of BCI, demonstrating the feasibility of using an in-ear electrode for collecting SSVEP potentials [34].

## 3. User Demand Exploration and Product Design Objectives

### 3.1. Design and Development Ideas

**Figure 1:**
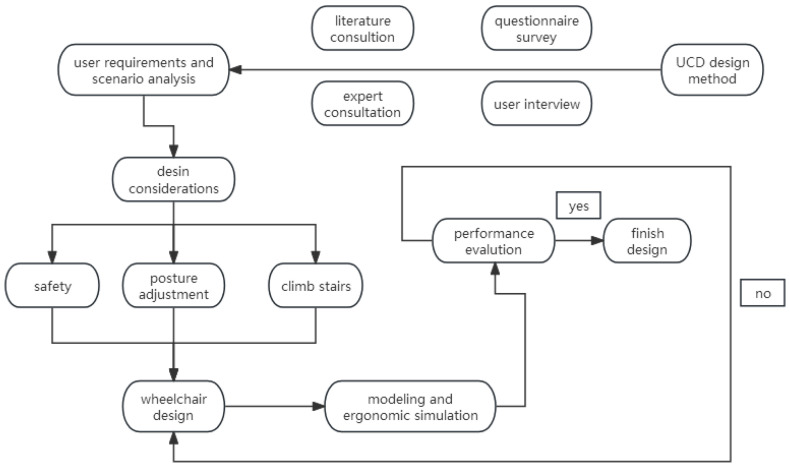
Design flow chart.

The starting point of this study was setting the aim to meet the actual needs of people with lower limb disabilities in intelligent wheelchairs. Based on the UCD concept, the demands of people with lower limb disabilities were summarized. Then, through a process of structure and function design, computer modeling, simulation, and so on, the product performance was evaluated according to simulation results, and the design was future improved based on the issues found. The detailed design flow chart is demonstrated in Figure 1.

### 3.2. Analysis of User Behavior Characteristics and Usage Scenarios

After establishing people with lower limb disabilities as the design target group, the behavioral and psychological characteristics of users were analyzed in detail through a literature review, questionnaire survey, and expert consulting. At the psychological level, some people with lower limb disabilities often feel pressure brought by the surrounding environment as they have to maintain a prolonged sitting position and look up when talking with others or observing the nearby environment. In addition, the excessive metal elements and exposed mechanical structures in common intelligent wheelchair products on the market make users feel indifferent. At the physiological level, more than half of the respondents noted that they live with very limited accessibility facilities; as a result, their daily travel is extremely complicated, and it is almost impossible for them to travel alone without the care of others. In addition, the unreasonable design of some intelligent wheelchair seats leads to physical problems in long-term use, such as lumbar muscle fatigue.

Based on the interviews with target users, combined with informal observation, a systematic analysis of the temporal and spatial working scenes of intelligent wheelchairs was carried out. In addition, the use habits of intelligent wheelchair products were also systematically studied. According to the analysis, it was found that intelligent wheelchairs are used at a high frequency and for a long time, which do not change with other conditions. The most common use scenario is an indoor environment such as the bedroom, followed by parks, squares, and other outdoor public leisure areas.

### 3.3. Requirements Evaluation of Function and Design Attributes

(1) Requirement research

According to the analysis of the user behavior characteristics and usage scenarios, the actual requirements of people with lower limb disabilities for intelligent wheelchair design were established. A total of 200 paper survey questionnaires were sent to a large number of people with lower limb disabilities, and 186 valid questionnaires were recovered. Based on the valid questionnaire data and interviews with individual lower limb disabled users, 15 function and design attributes of intelligent wheelchairs were summarized. Then, the frequency of each function and design attribute in different types of requirements was calculated using the Kano model.

The Kano model, proposed by Japanese scholar Nosho, is used to analyze the impact of user needs on satisfaction, and to classify and prioritize user needs, which reflects the non-linear relationship between product functional needs and user satisfaction [35]. M is the basic demand, where if the product has no such demand, the user will be greatly disappointed; O is the desired demand, where if the product can solve such demand, the user satisfaction will increase; A is the attractive demand, and when the product meets such demand, the user satisfaction will increase; I is the indifference demand, and whether the product meets such demand will not greatly affect the user satisfaction; Q indicates the doubtful demand, where when the product meets the demand, the user satisfaction is doubtful; and R indicates the reverse demand.

The results of the Kano model are demonstrated in Table 1. The posture adjustment, automatic stair climbing, and wheelchair safety and comfort have high M values, which belong to the basic requirements. The appearance design, elevation of wheelchair seats, and convenience for users to move between the bed and the wheelchair are class A exciting needs. If the structural design of the intelligent wheelchair is able to fulfill the above needs, then users will view it more favorably.

(2) Requirement analysis

Based on the survey results, the accuracy and effectiveness of the requirements were verified through on-site observation, communication, and user experience testing. By delving into the living environments of people with lower limb disabilities and engaging in on-site communication, it was found from the statements and observations of five patients that their main mobility aid is a regular wheelchair, which requires the assistance of others to move, placing additional pressure on family members. Some people with lower limb disabilities may choose to walk with crutches, but this method also has many drawbacks. They still need support from others when climbing stairs, and using crutches also poses high safety risks. Alternatively, some people with lower limb disabilities choose electric wheelchairs, but their control is not flexible and their functions are limited, and human–computer interaction is not ideal.

In addition, through experience testing of patients using intelligent wheelchairs, it was found that people with lower limb disabilities use intelligent wheelchairs frequently and for a long time. They spend most of the day using intelligent wheelchair products. Therefore, intelligent wheelchairs must meet basic needs such as sitting, moving, and lying down, and they must have safety and comfort. The most common usage scenario for intelligent wheelchairs is indoor environments, which means that the steering of intelligent wheelchairs must be flexible, with a small turning radius, and they must be able to adapt to narrow and crowded indoor environments.

From the above results, it could be inferred that most people with lower limb disabilities have a strong desire to use intelligent wheelchairs. The strong demand for intelligent wheelchair posture adjustment, automatic stair climbing, and wheelchair safety and comfort verified the accuracy and effectiveness of the survey questionnaire.

### 3.4. Product Design Objectives

In order to solve the current problem of intelligent wheelchair products in terms of ergonomics, a safe and efficient intelligent wheelchair capable of posture adjustment, stair climbing, and seat elevation was independently designed based on the results presented by the Kano model. In addition, it was required that the intelligent wheelchair also meet the aesthetic needs of users. Furthermore, this wheelchair was designed to allow for changes in sitting and lying positions, to overcome the bulkiness of existing tracked stair climbing wheelchairs, and with wheelchair seats and backrests based on ergonomic principles to address user comfort issues. Meanwhile, in order to meet the needs of individuals with mobility impairments for independent movement, this study aimed to design a brain–computer interface system based on SSVEP to achieve control over the movement of intelligent wheelchairs.

## 4. Design of a Brain-Controlled System for an Intelligent Wheelchair

Brain–computer interface (BCI) technology enables direct communication between the human brain and external devices, allowing individuals to interact with computers via brain signals. Traditional control methods for intelligent wheelchairs, such as buttons and joysticks, require some degree of physical mobility, which can be challenging or even impossible for individuals with motor function disabilities. In contrast, a brain-controlled wheelchair (BCW) bridges the brain and the wheelchair, offering direct and intentional control to those with motor function impairments [36].

### 4.1. Selection and Design of Brain–Computer Interface Modalities

According to the type of control signal, the commonly used intelligent brain-controlled wheelchair systems are categorized into sensorimotor rhythm (SMR)-based BCI, P300-based BCI, and steady-state visual evoked potential (SSVEP)-based BCI.

SMR, as an endogenous brain signal, mainly consists of mu and beta rhythms with frequency ranges of 7–13 Hz and 13–30 Hz, respectively. Event-related desynchronization (ERD) or event-related synchronization (ERS) will show in the SMR when subjects perform motor movement/imagery or rest. The SMR-based BCIs have distinct signal features for classification without an external stimulus but require long training times and can only offer limited control options [37].

P300 potentials are event-related potentials observed in EEGs at around 300 milliseconds post-stimulus. P300-based BCIs have the advantages of high accuracy and a short training time among most subjects, but they have relatively low information transfer rates and quickly lead to subject fatigue [38].

SSVEP signals are evoked when subjects gaze at a visual stimulus, which has peak responses at the frequency of the stimulus and its harmonics. The literature indicates that SSVEP signals have low noise and high reliability in the 6–30 Hz frequency range, and the minimal frequency resolution is 0.2 Hz. Despite the potential visual fatigue from long-term experiments, SSVEP-based BCIs, requiring no training, offer high accuracy, multiple commands, higher information transfer rates, and simple implementation, meaning they are becoming a hotspot in the BCI research field. Thus, this work utilized the SSVEP signal for the control signal of a brain-controlled wheelchair [39].

### 4.2. SSVEP-BCI System Framework

**Figure 2:**
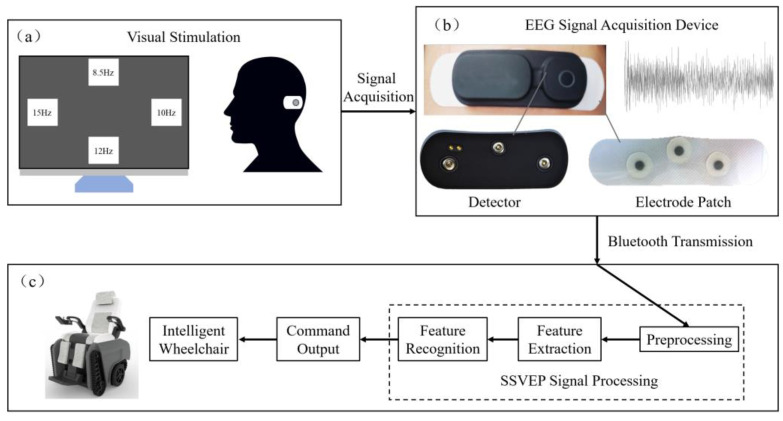
The framework of the proposed SSVEP-based brain-controlled wheelchair system. (**a**) Four visual stimuli are selected to construct a visual stimulation system. (**b**) A single channel EEG device is used to collect the subjects’ EEG signals. (**c**) The EEG signals is transmitted by Bluetooth to a computer to decode and output control commands for the intelligent wheelchair.

The framework of the proposed SSVEP-based brain-controlled wheelchair system is shown in Figure 2. The subject, who wears an EEG signal acquisition device, delivers control intention by focusing on visual stimuli on a monitor. The EEG signals are wirelessly transmitted to a computer via Bluetooth and are decoded into the control signals of the intelligent wheelchair.

### 4.3. Design of the Visual Stimulation System

The visual stimulation system displays stimuli of designated frequencies on the monitor to stimulate the subject’s visual nerves, which affects the BCI information throughput and the levels of user safety and comfort [40]. In this context, the following aspects need to be considered:

1. Visual Stimulation Device: A liquid crystal display (LCD) and light emitting diode (LED) are commonly used for displaying visual stimuli. While an LED can provide high luminance and accurate frequency, complex hardware is required to achieve precise flickering. An LCD is preferred here for its simplicity and the ability to generate stable visual stimuli via computer programming.
2. Visual Stimulation Methods: Pattern reversal and graphic stimulus alternation are the two main methods for visual stimulation. The former is achieved by repeatedly turning a rectangle or chessboard on and off at a fixed frequency, and the latter splits a rectangle into multiple parts and turns them off sequentially at a fixed frequency. The pattern reversal method was chosen due to the lower visual fatigue.
3. Visual Stimulation Frequency: SSVEP signals have the peak response at the stimulation frequency and its harmonics. SSVEP responses evoked by two different visual stimuli overlap when one stimulus’s stimulation frequency is multiple times the other. Moreover, the available frequencies are limited by the refresh rate of the monitor due to the black–white flickering modulation. The available frequencies of the 60 Hz monitor are shown in Table 2. Considering the abovementioned factors, 7.5 Hz, 10 Hz, 12 Hz, and 15 Hz were finally selected for stimulation.

The graphic user interface (GUI) of the visual stimulation system is shown in Figure 2a. The resolution of the monitor is 1920 × 1080. Four visual stimuli are located at the monitor’s up, down, left, and right, with the size of 200 × 200 pixels, indicating forward, backward, left turn, and right turn commands. Four visual stimuli were repeatedly designed as rectangles flickering at the designated frequencies from black to white.

### 4.4. EEG Signal Acquisition and Preprocessing

The existing EEG signal acquisition devices primarily collect signals from multiple channels, which makes them expensive and heavy, and they require sophisticated preparation for wearing and cause discomfort. Thus, they are unsuitable for long-term use in the brain-controlled wheelchair system. This work used a single-channel EEG acquisition device, as shown in Figure 2b. The device can be easily stuck onto the occipital region and is lightweight, small, easy to use, and comfortable. The gel electrode is used to stick onto the skin with a low impedance and high signal-to-noise rate (SNR). The center electrode is the reference channel, and the two electrodes on both sides are for EEG signal acquisition. The device collects EEG signals and transmits them to a computer for processing wirelessly through Bluetooth, with a sampling rate of 250 Hz.

**Figure 3:**
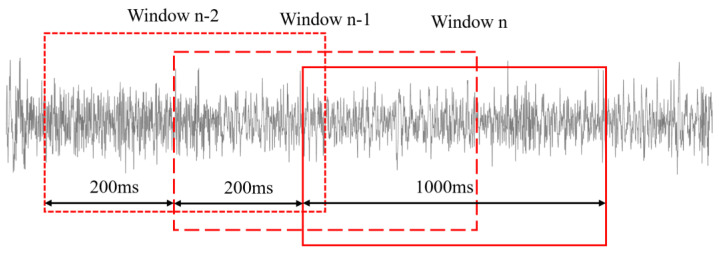
The sliding window strategy.

After the computer receives the raw EEG signal, preprocessing is required to increase the SNR. The notch filter is used to eliminate the power noise, and a 2-order Butterworth bandpass filter with a passband of 0.5–45 Hz is used to filter out the noise of low and high frequencies. Figure 3 shows the EEG signals after preprocessing, and the clear waveform indicates the high quality of the EEG signals. Considering the real-time requirement of the decoding algorithm for controlling the intelligent wheelchair, the sliding window strategy is utilized to process the EEG signals. Overlapping EEG signals of 1 s with a step of 200 ms are extracted for further SSVEP decoding.

### 4.5. SSVEP Recognition Algorithm

**Figure 4:**
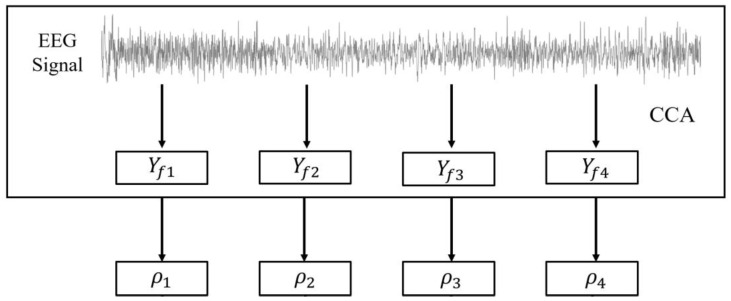
The scheme for CCA-based SSVEP decoding.

Canonical correlation analysis (CCA) is a multivariate statistical method that calculates the underlying correlation between two multi-dimensional random variables. CCA is widely used for SSVEP decoding due to its high precision, low computational complexity, and ease of use [41]. The SSVEP decoding process using CCA is shown in Figure 4.

Y represents the reference signal for each class, containing the frequency information of the stimulation frequency and its harmonics. The reference signal $Y_{f}$ could be defined as

(1) $$Yf=\left[\begin{array}{c} \sin(2\pi ft)\\ \cos(2\pi ft)\\ •\\ •\\ \sin(2\pi Nft)\\ \cos(2\pi Nft) \end{array}\right]$$

where N is the harmonic number and equals four. f is the stimulation frequency, which is 7.5 Hz, 10 Hz, 12 Hz, or 15 Hz for each stimulus, respectively. The correlation between the test sample and the reference signal of each stimulus is calculated. Due to certain features of the spectrum, to improve the classification performance, the signals are subjected to Fast Fourier Transform (FFT) to obtain the amplitudes of the spectrum at 7.5 Hz, 10 Hz, 12 Hz, and 15 Hz, which are then used as features for classification training.

This work used Support Vector Machines (SVMs) for data classification, with the kernel function chosen as a third-degree polynomial kernel. SVM was used as the classification algorithm to perform five-class classification on EEG signals captured during gaze at four flickering blocks with different frequencies and rest. SVM is a supervised machine learning algorithm used for classification and regression tasks, aiming to find an optimal hyperplane that maximizes the margin between different classes in the feature space.

Finally, the target was mapped to the control command of the intelligent wheelchair. In doing so, the brain-controlled wheelchair BCI system setup was completed.

### 4.6. System Performance Validation

**Figure 5:**
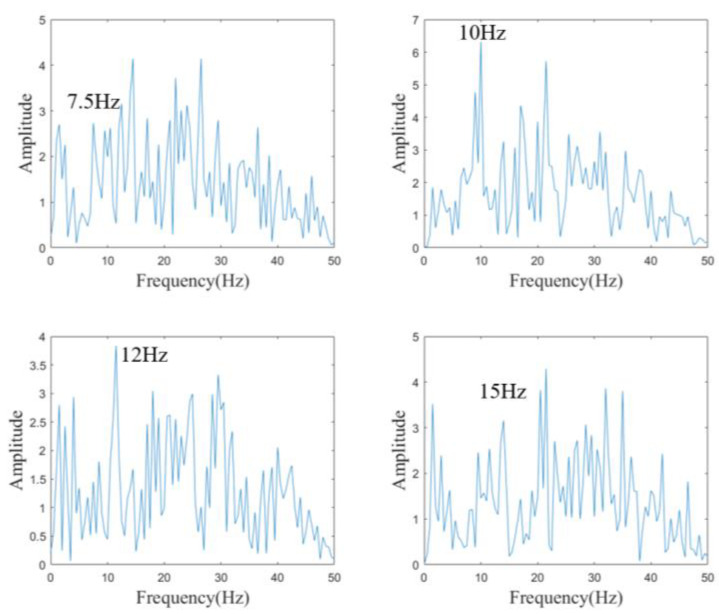
SSVEP feature obtained with FFT.

During the experiment, a subject is asked to focus on each of the fixation targets and EEG data are collected for 20 s at each of the fixation targets. Between each session of gazing at the flickering blocks, the subject rests with their eyes closed for two minutes to prevent visual fatigue and preserve signal quality. Figure 5 shows the features obtained with FFT of the collected EEG signals at each frequency. As can be seen from the spectra, the peak is obtained at the corresponding frequency. Due to the presence of spontaneous brain electrical activity, the peaks are not very prominent, but the spectra still contain the required features.

**Figure 6:**
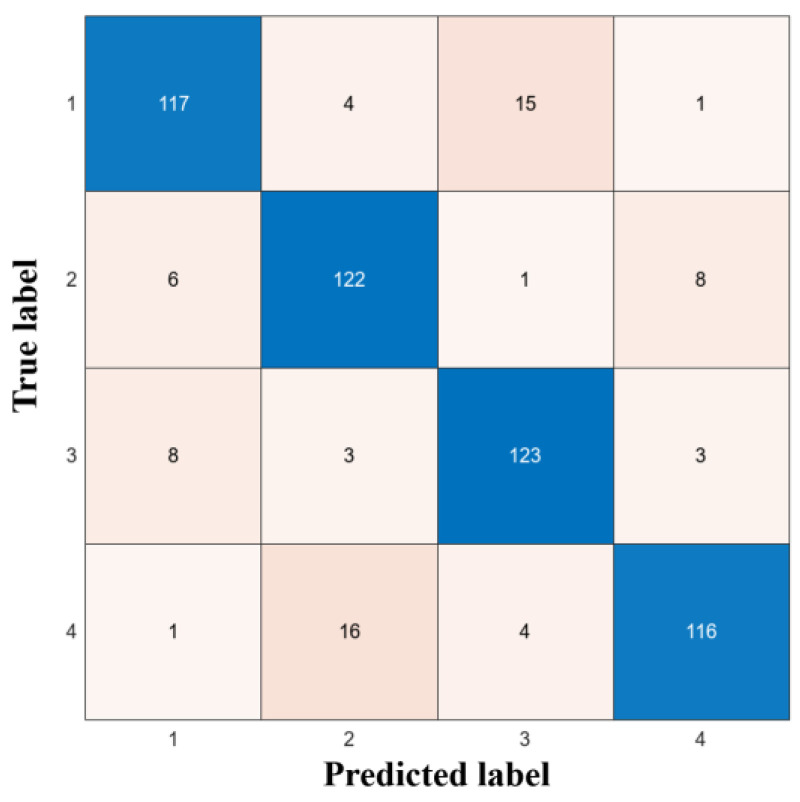
Confusion matrix.

The dataset was divided as 80% training set and 20% validation set. After classification, the confusion matrix was obtained, as shown in Figure 6, with a classification accuracy of 83.1%. The experimental results show that the system has a good classification performance and meets the usage requirements.

## 5. Research on Ergonomics and Intelligent Wheelchair Seat Design

### 5.1. Ergonomic Design Philosophy

Ergonomics is an interdisciplinary subject of psychology, biology, metrology, human anatomy, and other disciplines, which pursues the optimality of the whole human–machine–environment system, rather than an individual element [42]. In addition, as the intelligent wheelchair is classified as a national class I product, the seat design, seat size, and other aspects need to be guided by the three principles of ergonomics.

1. Safety principle

Safety and reliability are the basic points of product design and also the focuses of ergonomics. If the safety of the intelligent wheelchair cannot be guaranteed, users are prone to safety risks such as rollover in the process of use. Therefore, the structure of the intelligent wheelchair should be reasonably designed to ensure stability during stair climbing and seat elevation. In the shell design of the intelligent wheelchair, round corner transitions should be used as far as possible to avoid damage caused by sharp edges and corners. The physical structure should have the function of restricting wrong and dangerous operation.

2. Comfort principle

The comfort of the intelligent wheelchair should be considered in the early stage of product design. People with lower limb disability are in a long-term sitting posture. If the seat design of the intelligent wheelchair cannot guarantee comfort, it is likely to cause the user a lumbar injury.

3. Generality principle

A people orientation is an important concept conveyed by ergonomics. The design of any product should be adapted to people’s use habits. Therefore, in the design process of an intelligent wheelchair, the human body structure and use habits should be fully considered to meet the requirements of the majority of users as far as possible.

### 5.2. Size Design of Intelligent Wheelchair

Intelligent wheelchairs belong to the general industrial type I products. According to the selection criteria of the percentiles of human body size, the size range of intelligent wheelchair seats is defined as the 5th percentile of the female body to the 95th percentile of the male body. When using intelligent wheelchair products, users are mainly in a sitting position. Therefore, the size parameters of intelligent wheelchairs mainly refer to the size data of various parts of the body in a sitting position, as shown in Table 3. The overall size design of the human body should consider the universality of the product as well as the physiological and psychological characteristics of the human body [42]. Therefore, it is necessary to add correction values for normal human clothing (Table 4) on the basis of the original standard values, in order to systematically analyze and design the size of intelligent wheelchairs based on standard values for the final calculated size.

#### 5.2.1. Design of Intelligent Wheelchair Seat Height

The seat height dimension refers to the vertical distance between the seat cushion of the intelligent wheelchair and the ground. When the human body is in a normal sitting position, the thighs are approximately level with the seat cushion, the calves naturally hang down, and the soles of the feet are flat on the ground. The size of the sitting height is related to parameters such as the size of calves plus the full height and the amount of clothing and body shape correction. To reflect the applicability design principles of the intelligent wheelchair product, the size of the tibial height was determined to be 382 mm in the 50th percentile for females. Following corrections for the dressed figure and the height of the intelligent wheelchair transmission system, we set the height dimension of the intelligent wheelchair seat to 515 mm.

#### 5.2.2. Design of Intelligent Wheelchair Seat Width

Seat width refers to the distance from the left end to the right end of the seat cushion of an intelligent wheelchair seat. The selection of sitting width should refer to the distance between the buttocks or thighs of people with lower limb disabilities when they are in a sitting position. Based on dressing correction and the psychological needs of people with lower limb disabilities, the sitting width requires a greater distance than the sitting hip width. According to the body size data in Table 3, it can be seen that the hip width of women in sitting positions is generally larger than that of men. Therefore, we used the 95th percentile hip width of women in the human body size table as the standard, and after adding clothing correction and psychological characteristics to the size, the sitting width size was determined to be 430 mm.

#### 5.2.3. Design of Intelligent Wheelchair Seat Depth

Seat depth refers to the distance between the front and rear ends of the seat cushion. The setting of size data should consider the person’s sitting depth, and the appropriate chair surface depth should provide sufficient support for the buttocks and allow the waist to be supported by the backrest. In addition, the front end of the seat should be kept at a distance from the calves, which can avoid thigh compression and allow the calves to move freely. In order to avoid compression on the backs of the calves for users with smaller body sizes, the female fifth percentile was used as the size standard and clothing correction values were added. The sitting depth was determined to be 410 mm.

#### 5.2.4. Design of the Intelligent Wheelchair Armrest Size

The height of the armrest refers to the vertical distance between the human elbow and the chair surface. According to the criteria for percentile selection and the height of the elbow in the sitting position, the 50th percentile value for males was selected as the height of the armrest. Considering the needs of decoration, the inclination angle between the backrest and the chair surface of the intelligent wheelchair, and the comfort of natural sagging of the human arm, the armrest height is usually chosen as lower than the sitting elbow height, so the armrest height was determined to be 250 mm.

#### 5.2.5. Design of the Intelligent Wheelchair Pedal Size

Based on the 95th percentile foot length and foot width dimensions for males in the human body size table, plus correction, the pedal length dimension was obtained as 300 mm. The width dimension of the pedal was taken as 120 mm. When using an intelligent wheelchair, people with lower limb disabilities should sit in a sitting position, and the distance between their feet should be slightly less than their shoulder width, so the distance between the pedals was set to 310 mm.

#### 5.2.6. Design of Intelligent Wheelchair Backrest Size

The height of the seat backrest refers to the distance from the upper end to the lower end of the backrest. The selection of the backrest height mainly refers to the cervical height in the human sitting posture. Given the adjustable height headrest installed above the backrest of the intelligent wheelchair in this case, and the inclination angle between the backrest and the cushion, it was deemed that the height of the backrest should be less than the height of the human sitting posture. Based on the human body size and size correction values, we determined the seat back height to be 650 mm. In addition, the backrest of the intelligent wheelchair should be supported at the waist to ensure that the curvature of the human spine is in a natural posture. Accordingly, the center position of the lumbar spine was approximately 165–210 mm above the backrest of the seat, and the lumbar spine fulcrum was slightly higher than this height. The height of the lumbar support must be at least equal to the height of the lumbar support point to support the weight of the back, so the height of the lumbar support was 220 mm above the wheelchair seat surface.

#### 5.2.7. Design of Intelligent Wheelchair Chair Tilt Angle

When designing seat cushions, it is necessary to increase the inclination angle of the chair surface to further enhance the comfort of using intelligent wheelchairs. The inclination angle of the seat surface refers to the angle between the seat cushion and the horizontal plane. Generally, the seat cushion needs to be slightly tilted backwards, which brings two benefits. Firstly, it can increase the stability of driving intelligent wheelchairs for people with lower limb disabilities. Secondly, through the action of gravity, the backs of people with lower limb disabilities can fit more tightly with the backrest of the seat, providing better support for the body. However, the inclination angle of the chair cannot be too large. If the inclination angle of the chair is too large, it will compress the abdomen of the human body. Therefore, the inclination angle of the chair surface should be moderate. In this study, according to the value rules of the inclination angle of the chair surface, the inclination angle of the chair surface was determined to be 15 degrees.

### 5.3. Intelligent Wheelchair Seat Surface and Backrest Design

Intelligent wheelchair seat surface design needs to comply with the body posture. It is known from the human anatomy that numerous nerves and blood vessels are distributed in the human crotch area, and when excessive pressure is applied to this area, the blood circulation and nerve conduction will be compressed. Therefore, the seat surface should be designed according to the pressure characteristics under different hip areas; namely, when the sciatic pressure is increased, the pressure is gradually reduced around [43]. Accordingly, the seat surface of the intelligent wheelchair was designed to adopt a curved shape corresponding to the shape of the hip, and the seat surface under the ischial tuberosity was almost horizontal. Memory cotton material was used to increase elasticity and further improve the seat comfort.

The backrest design of the intelligent wheelchair followed the shape of the human spine. Firstly, it was necessary to clear the pressure applied to the backrest when the human body was sat up with a straight posture. The lumbar support provided major support, while the shoulder support was not stressed. When the human body is relaxed, the spine presents a natural physiological curvature, and the whole body is in a comfortable rest position. In order to maintain a sitting posture with a normal lumbar curve, there should be about 105-degree angle between the trunk and the thigh, along with some support at the waist. In this case, the central position of the lumbar spine was about 165~210 mm above the seat, and the lumbar fulcrum was slightly higher. The height of the lumbar support should be at least equal to the height of the lumbar fulcrum to support the weight of the back, so the height of the lumbar support was set at 220 mm above the wheelchair seat [44].

## 6. A New Intelligent Wheelchair Design Practice

### 6.1. Determination of the Design Scheme

**Figure 7:**
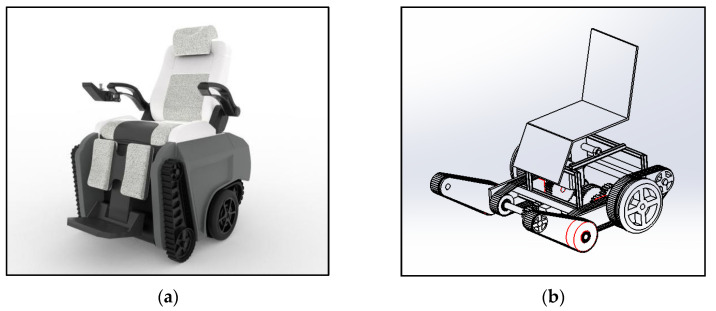
Intelligent wheelchair rendering and structural schematic diagram. (**a**) Smart wheelchair rendering. (**b**) Schematic diagram of intelligent wheelchair structure.

Based on the analysis of previous studies and questionnaire survey data, by combining ergonomics, user behavior modes, product use environments, etc., the dimensions and functions of the intelligent wheelchair were determined, and the appearance and structural design of the intelligent wheelchair were completed, as shown in Figure 7.

In this design, the seat and backrest are structured according to the ergonomic size. The special curved surface of the backrest can fit the human spine well and increase the comfort in the back of a user with lower limb disabilities. In addition, the tilt angle between the seat and the backrest can also be adjusted according to the needs of users. The transmission scheme of the wheelchair adopts a combination of segmented tracks and tires to ensure safety in stair climbing and to increase the flexibility in steering.

### 6.2. Description of the Functional Design

#### 6.2.1. Intelligent Wheelchair Lift Mechanism

**Figure 8:**
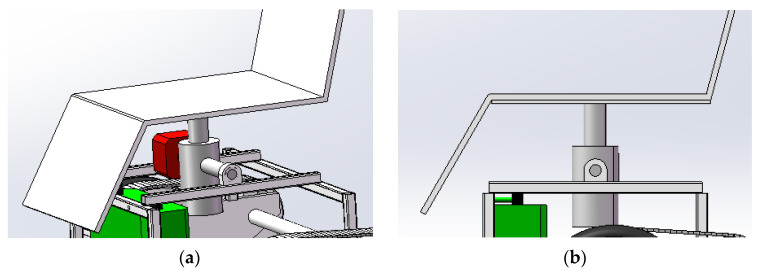
The structure of the lifting device. (**a**) Assembly diagram of seat lifting mechanism. (**b**) Side view of seat lifting mechanism.

**Figure 9:**
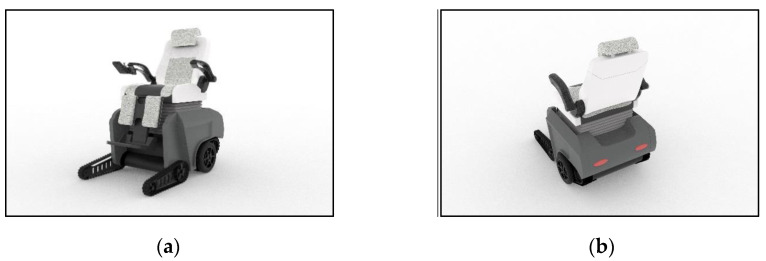
Seat lifting effect diagram of the intelligent wheelchair. (**a**) The front view of seat lifting effect diagram. (**b**) The rear view of seat lifting effect diagram.

The seat lift mechanism of the intelligent wheelchair is mainly realized through an electric lifting device [45]. The electric lifting mechanism is shown in Figure 8. The seat-lifting electric push rod in Figure 8a is used to push the seat up and down, and the horizontal angle of the seat is adjusted by balancing the motor; Figure 8b shows a side view of the seat-lifting mechanism. When climbing the stairs, the horizontal angle of the wheelchair is ensured through the collaborative work of the gradienter and the gyroscope. In order to prevent a center-of-gravity shift during lifting caused by the small action area of the lifting rod [46], a folding tent car block is installed at the connection area between the seat and the frame to stabilize the center of gravity of the seat and increase the cushioning of the wheelchair seat when the intelligent wheelchair is driven along a rough roadway. A seat-lifting effect diagram is shown in Figure 9.

#### 6.2.2. Multi-Attitude Adjustment of Intelligent Wheelchair

**Figure 10:**
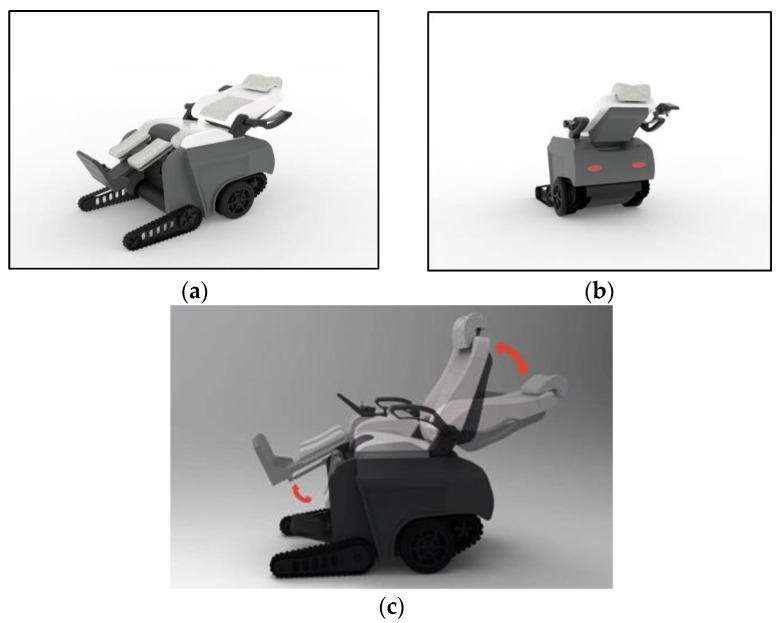
Lie down effect diagram of intelligent wheelchair. (**a**) The front view of intelligent wheelchair lying down effect diagram. (**b**) The rear view of intelligent wheelchair lying down effect diagram. (**c**) Schematic diagram of intelligent wheelchair posture transformation.

The maximum angle between the seat and the backrest can be achieved by adjusting the knob between the seat and the backrest. At the same time, the wheelchair seat lifts to change the entire wheelchair into a lying down state. Through the combination of the electric lift device and the adjustment device, which control the seat and backrest, a set of wheelchair lying posture schemes within the range of 30° forward and 20° backward angles is made possible in this design practice of the intelligent wheelchair. The lying down state of the intelligent wheelchair is shown in Figure 10.

In this state, the height of the intelligent wheelchair seat is close to the height of the bed. Then, users with lower limb disabilities can easily lift the armrest on either side and manage to move from the wheelchair to the bed.

#### 6.2.3. Intelligent Wheelchair Stair Climbing Mechanism

At this stage, according to the operating principle of stair climbing, in addition to structural schemes that use various auxiliary mechanisms, the three main structural modes include leg foot, wheelchair back crawler, and wheelchair bottom crawler [47].

The leg-foot climbing mechanism applies the hinge rod device, which was developed in the early stage of the market. Stair climbing is realized through alternating movements of the robotic arms and moving forward horizontally. The process of stair climbing is the repetition of these two movements, which is basically an imitation of the process of human stair climbing [48]. From the perspective of its mechanism, the leg and foot climbing structure has the advantage of flexibility. However, the main disadvantage of this structural mode is that the hinge rod device of the leg-foot climbing structure is complex and likely to cause instability of the center of gravity and a shake of the seat during climbing.

At the present stage, the crawler climbing structure is relatively mature and widely used. Whether it is the crawler climbing mechanism on the back or the bottom of the wheelchair, its principle is similar to that of tracked armored vehicles. When moving on the stairs, its center of gravity will always be parallel to the edge of the stair steps, so that the fluctuation of the center of gravity of the wheelchair is reduced to achieve smooth movement [47]. Due to the large contact area between the track and the road surface and small pressure, this mechanism shows good obstacle crossing and slope climbing performance, and it is able to pass through various complex terrains.

**Figure 11:**
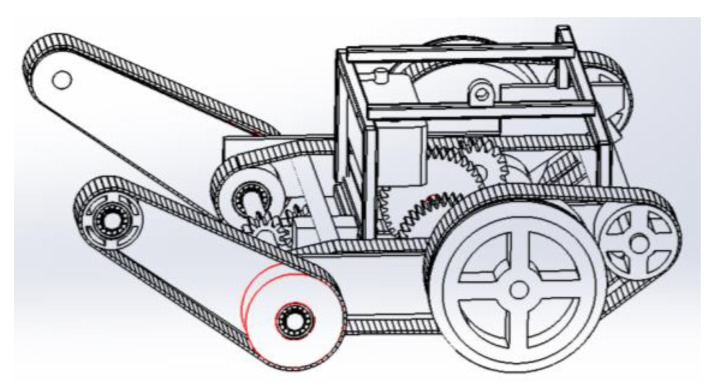
Segmented crawler climbing mechanism.

The main disadvantages of traditional wheelchair mechanisms include slow driving, large turning radii, and difficulty turning, which lead to poor flexibility. Therefore, after comprehensively considering factors such as practicability, design difficulty, and the cost of the wheelchair, a new segmented track mechanism was used in this design, which divides the original two long track structures into two-stage track structures, as shown in Figure 11. The segmented track structure reduces the radius of rotation of the intelligent wheelchair and makes it easier for the intelligent wheelchair to adjust its angle when climbing stairs, which can help to maintain its stability. A tire is added to the design of the rear track. The tire provides better damping and greater traction for the track, and it further assists the wheelchair track to conduct steering.

The intelligent wheelchair operation in this design practice is mainly through the combination of four parts: joystick, wheelchair controller, automatic cruise module and mobile phone connection module.

The joystick is located on the right side of the wheelchair armrest, under the mobile phone wheelchair connection device, serving to control the direction, speed, etc. A push forward represents the forward movement of the wheelchair, and the user pushes backward or presses the emergency button on the control button to brake. The shape of the joystick coincides with the relaxed posture of the hand. Liquid silicone is used on the surface material of the joystick, which is soft, skin-friendly, safe, and non-toxic.

The controller is located on the left side of the armrest of the wheelchair. For the convenience of people with lower limb disabilities, each button is marked with different color icons and text.

In addition, wave radar is added above the baffle of the front and rear tracks of the intelligent wheelchair to ensure the accurate perception of the peripheral environment. In the state of autonomous driving, the mobile phone is connected with the forward monocular camera and the backward radar sensor for remote measurement to form a panoramic model.

## 7. Ergonomic Simulation Research

### 7.1. Data of Human Model and Standing Posture Simulation

**Figure 12:**
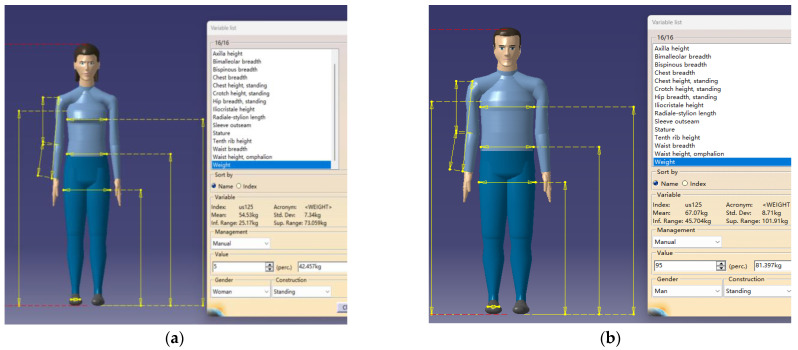
Human body model based on CATIA. (**a**) Female P5 percentile human model. (**b**) Male P95 percentile human model.

We used the Human Builder section of the CATIA ergonomics simulation module to create two human models. We selected China as the country option for the human body model, and in order to test the universality of the smart wheelchair, we established a Chinese male human body model with a percentile of P95 and a Chinese female human body model with a percentile of P5. The size data of the human body model are shown in Figure 12, and the dimensions of various parts of the body, mainly including the chest, waist, head, and shoulders, were obtained from the model.

### 7.2. Movement Analysis of Human Model

**Figure 13:**
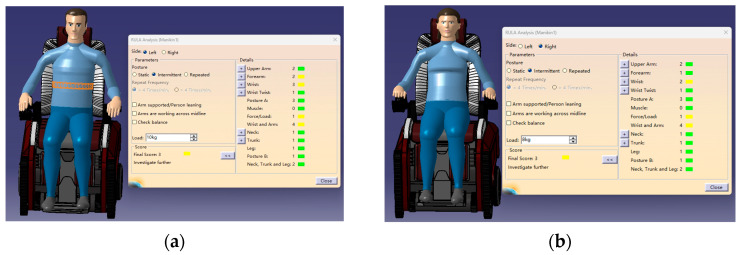
Movement analysis of human model. (**a**) Results of male exercise analysis. (**b**) Results of female exercise analysis.

Our motion simulation was a simulation analysis of the movement state of the individual with the product in a standing posture. The data presented by the movement analysis module were used to determine whether the individuals felt uncomfortable when the wheelchair was moving. The comfort levels of the human body during the movement are representatively marked by four different colors, where green represents 1–2 points, indicating that the product is relatively comfortable in this state; yellow represents 3–4 points, indicating that the corresponding position needs minor adjustments; orange is 5–6 points, indicating that the corresponding position is acceptable [49]; and red is 7 points, indicating that the posture causes fatigue and extreme discomfort, meaning that it needs to be changed immediately, as shown in Figure 13.

### 7.3. Sitting Posture Simulation of the Human Body Model

**Figure 14:**
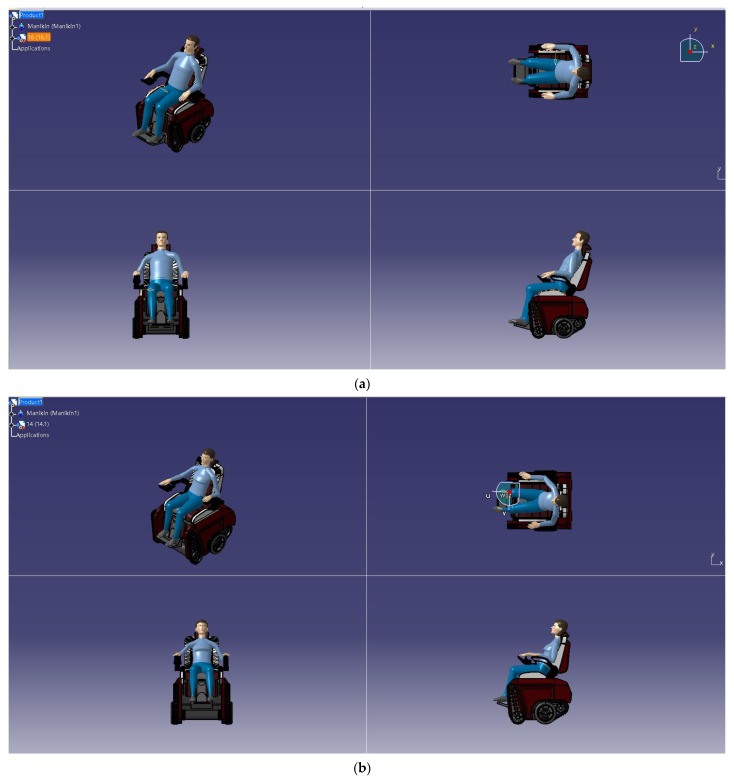
Sitting posture diagram of human body model. (**a**) Simulation of sitting posture of male human body model. (**b**) Simulation of sitting posture of female human body model.

A simulation of the human body and the product in a sitting posture was also carried out. The pose editing module in CATIA (V5 r21) software was used to adjust the data of the human body in the sitting position, such as the values of the buttocks, spine, and waist. Then, a combination of the human body model and product model was realized through the Reachmode contact command, to simulate different sitting postures in product use [50], and we conducted an interactive analysis between the human body model and intelligent wheelchair model (as shown in Figure 14).

### 7.4. Analysis of Sitting Posture Comfort

**Figure 15:**
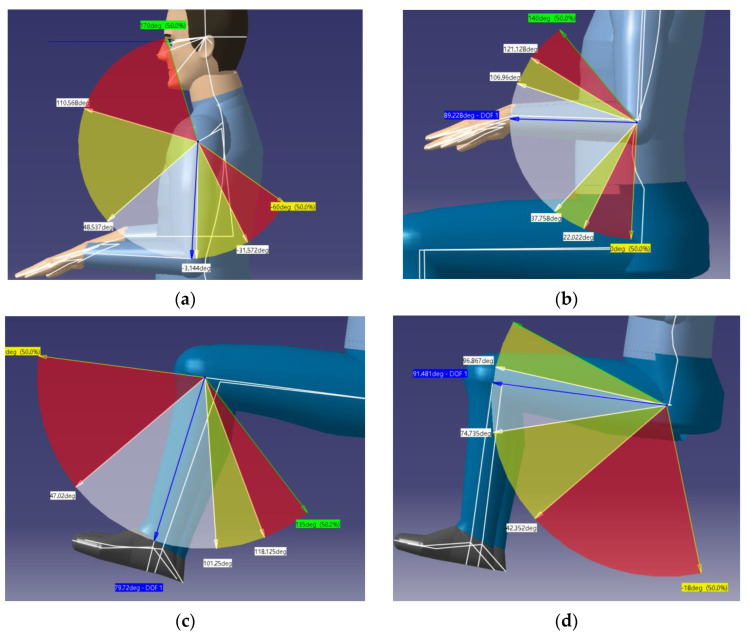
Ranges of motion of various parts of the human body. (**a**) Schematic diagram of upper arm range of motion. (**b**) Schematic diagram of forearm range of motion. (**c**) Schematic diagram of calf range of motion. (**d**) Schematic diagram of thigh range of motion.

The evaluation of product comfort was mainly based on factors such as human posture and exercise behavior. With the help of CATIA’s human posture analysis tool, the comfort of intelligent wheelchairs can be evaluated. During the process of performing human pose analysis, the Human Posture Analysis module of the software is used to set angle constraints and select preset parameters, in order to accurately simulate various joint degrees of freedom and ranges of motion of the human body, as shown in Figure 15. Among them, the blue arrow represents the range that can be used for product adjustment, the green arrow and yellow arrow represent the extreme positions of each part, and the semitransparent area is the range that various parts of the human body can move in. Here, the percentages of the CATIA posture analysis are shown in three colors: red, yellow, and white, with the white area set as the most comfortable area for the human posture; the yellow area set as a generally comfortable area for the human posture; and the red area referring to the uncomfortable area for the human body posture, which can cause muscle fatigue if maintained for a long time.

**Figure 16:**
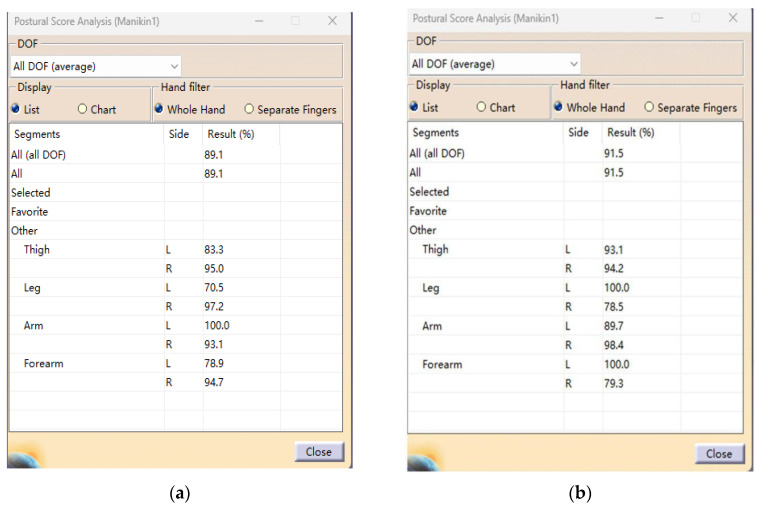
Human model comfort report. (**a**) Report on comfort of male human model. (**b**) Report on comfort of female human model.

We used posture evaluation tools in the software to measure the sitting postures of simulated humans, and we generated a posture comfort analysis report (Figure 16), where the specific scoring data are expressed in percentages. The larger the number, the more comfortable the human posture. The average comfort level of each part of the human model was 89.1% in one case and 91.5% in the other.

In the above figure, it can be seen that the two human models at different percentiles feel comfortable overall, and this design basically meets the requirements of ergonomics for the product.

## 8. Conclusions

First of all, the current research status of intelligent wheelchair products at home and abroad was investigated through literature research to understand the latest functions, structures, and design methods of intelligent wheelchairs. Secondly, taking users with lower limb disabilities as the design focus, behavioral characteristics and usage scenarios were analyzed through a questionnaire survey, user interviews, expert consultation, and informal observation. Subsequently, the function and design attribute requirements of users for intelligent wheelchair products were analyzed, and the product design targets were defined. Subsequently, to achieve brain-controlled intelligent wheelchairs, a brain–computer interface system based on SSVEP was designed. Finally, intelligent wheelchair products were upgraded in their structure, size, and control mode. The designed intelligent wheelchair product is safe, efficient, convenient to use, and has complete functions in line with the UCD and ergonomic design concept, which provides a reference for the upgrading of related intelligent wheelchair products. Nonetheless, although this study has achieved some results, there are still limitations that require further research and improvement. For example, quantitative and qualitative analyses of the safety, efficiency, and usability of intelligent wheelchair products were not conducted in actual usage scenarios. We plan to conduct relevant research in future work.

With a view to the future, we see that the development of artificial intelligence technology, the control methods of intelligent wheelchairs will become more diversified, and through deep learning, these chairs will begin to understand a user’s usage habits and automatically provide corresponding services for the user. In line with this, according to the different needs of users, intelligent wheelchairs will develop in the direction of high-end customization.

## Figures and Tables

**Table 1:** Kano model of function and design attribute evaluation.

Function and Design Properties	Types of Requirements						Evaluation Results
	M	O	A	I	Q	R	
Wheelchair exterior design	15	4	68	13	0	0	A
Wheelchair posture adjustment	72	11	15	2	0	0	M
Automatically travels up and down the stairs	90	4	6	0	0	0	M
Style/fashion	22	54	16	5	3	0	O
Night illumination	17	21	16	43	3	0	I
Mobile terminal control	14	8	18	52	4	0	I
Easy mobility to and from bed	32	0	68	0	0	0	A
Wheelchair seats raised	32	12	56	0	0	0	A
User body data sharing	0	0	1	23	4	72	R
Long single working hours	27	54	16	3	0	0	O
Quick charge	30	48	15	7	0	0	O
Safe and efficient	97	1	2	0	0	0	M
Wheelchair entertainment function	1	0	0	20	11	68	R
Wheelchair e-book	0	5	20	21	8	46	R
Wheelchair color scheme of choice	10	1	13	67	9	0	I

Sample: attributes are measured in % in the table: M—basic requirements; O—expectation type demand; A—exciting demand; I—no difference demand; Q—doubtful problem demand; R—reverse demand.

**Table 2:** The available stimulation frequencies for a 60 Hz monitor.

**f**	f/2	f/3	f/4	f/5	f/6	f/7	f/8	f/9	f/10	f/11	f/12
**Hz**	30	20	15	12	10	8.57	7.5	6.7	6	5.45	5

**Table 3:** Human body size percentiles (sitting posture).

Category	Male						Female					
Percentile	1	5	50	90	95	99	1	5	50	90	95	99
Cervical height	599	615	657	691	701	719	563	579	617	648	657	675
Shoulder height	539	557	598	631	641	659	504	518	556	585	594	609
Knee height	441	456	493	523	532	549	410	424	458	485	493	507
Elbow height	214	228	263	291	298	312	201	215	251	277	284	299
Seat depth	407	421	457	486	494	510	388	401	433	461	469	485
Hip width	284	295	321	347	355	369	295	310	344	374	382	400
Tibial height	372	383	413	439	448	463	331	342	382	399	405	417
Foot width	82	85	94	100	101	103	75	79	86	91	93	96
Foot length	224	232	250	261	269	278	208	215	230	243	247	256

**Table 4:** Dressed figure correction value.

Subject	Size Correction Value	Reason	Subject	Size Correction Value	Reason
Sitting height	3 mm	trouser thickness	Chest width	8 mm	clothes
Hip width	13 mm	clothes	Shoulder width	13 mm	clothes
Foot length	30–38 mm	shoes	Leg thickness	13 mm	clothes

## Data Availability

Data are contained within the article.
